# A Modular Experimentation Methodology for 5G Deployments: The 5GENESIS Approach

**DOI:** 10.3390/s20226652

**Published:** 2020-11-20

**Authors:** Almudena Díaz Zayas, Giuseppe Caso, Özgü Alay, Pedro Merino, Anna Brunstrom, Dimitris Tsolkas, Harilaos Koumaras

**Affiliations:** 1 ITIS Software, Universidad de Málaga, Andalucía Tech, 29071 Málaga, Spain; pmerino@uma.es; 2 Simula Metropolitan Center for Digital Engineering, Pilestredet 52, 0167 Oslo, Norway; giuseppe@simula.no (G.C.); ozgu@simula.no (Ö.A.); 3 Deparment of Informatics, University of Oslo, 0315 Oslo, Norway; 4 Deparment of Mathematics and Computer Science, Karlstad University, 651 88 Karlstad, Sweden; anna.brunstrom@kau.se; 5 Fogus Innovations & Services, 161 21 Kesariani, Greece; dtsolkas@fogus.gr; 6 NCSR Demokritos, Institute of Informatics and Telecommunications, 153 41 Paraskevi, Greece; koumaras@iit.demokritos.gr

**Keywords:** 5G, testbeds, experimentation, methodology

## Abstract

The high heterogeneity of 5G use cases requires the extension of the traditional per-component testing procedures provided by certification organizations, in order to devise and incorporate methodologies that cover the testing requirements from vertical applications and services. In this paper, we introduce an experimentation methodology that is defined in the context of the 5GENESIS project, which aims at enabling both the testing of network components and validation of E2E KPIs. The most important contributions of this methodology are its modularity and flexibility, as well as the open-source software that was developed for its application, which enable lightweight adoption of the methodology in any 5G testbed. We also demonstrate how the methodology can be used, by executing and analyzing different experiments in a 5G Non-Standalone (NSA) deployment at the University of Malaga. The key findings of the paper are an initial 5G performance assessment and KPI analysis and the detection of under-performance issues at the application level. Those findings highlight the need for reliable testing and validation procedures towards a fair benchmarking of generic 5G services and applications.

## 1. Introduction

The 5th Generation of mobile networks (5G) enables innovative use cases and services that provide the over the top service providers (a.k.a, verticals) with unprecedented performance capabilities. Indeed, it is expected that the 5G performance will span over the three extremes of bandwidth, latency, and capacity requirements, which enable enhanced Mobile Broadband (eMBB), Ultra Reliable and Low Latency Communication (URLLC), and massive Machine Type Communication (mMTC), respectively [1]. This potential is supported by a variety of technologies, including the Software-Defined Networking (SDN), the Network Function Virtualization (NFV), the Network Slicing, and the Multi-access Edge Computing (MEC). However, the quantification of 5G performance in an end-to-end (E2E) level is still ongoing, since the high heterogeneity of the enabled use cases requires various Key Performance Indicators (KPIs) and different target values [2,3]. Irrefutable, vertical- and use case-oriented 5G testing methodologies, and experimentation processes are required, which have to incorporate E2E aspects, far beyond the conventional component-oriented testing (which, so far, is mainly focused the radio access network [4]).

In this context, huge efforts are being made worldwide by 5G standardization, research, and stakeholder communities towards the implementation of experimental testbeds, where 5G technologies and KPIs can be reliably measured and validated in heterogeneous scenarios. Such testbeds should be flexible and automatically reconfigurable, employ different technologies (from early stage standardization to commercial components), and be able to reproduce several network conditions. This would enable common procedures for a fully automated experimentation, following a Testing as a Service (TaaS) paradigm [3]. To this end, EU and US programs, such as 5G Public Private Partnership (5G-PPP) (https://5g-ppp.eu (accessed on 30 August 2020).) and Platforms for Advanced Wireless Research (PAWR) (https://advancedwireless.org (accessed on 30 August 2020).), are specifically focusing on the realization of 5G testing platforms, aiming at providing trustworthy environments, where eMBB, URLLC, and mMTC verticals can quantify the expected performance of their services and the corresponding KPIs.

As part of the 5G-PPP initiative, the EU-funded 5GENESIS project (https://5genesis.eu (accessed on 30 August 2020)) is realizing a 5G facility composed of five different testbeds in Europe, accessible for both per-component and E2E experimentation purposes [5]. A reference architecture for common and lightweight access to the 5GENESIS testbeds has been already defined [6], along with an E2E methodology for testing and validation of 5G technologies and KPIs. More precisely, the 5GENESIS testing methodology follows a modular approach and it includes three logical components, referring to three configuration/input information classes required for running an experiment, namely, the test cases, the scenarios, and the slices. Altogether, they identify an experiment, the definition of which is also formalized in a global template, referred to as Experiment Descriptor (ED). From the architectural point of view, the information enclosed in the ED feeds a core functional block of the 5GENESIS reference architecture, the Experiment Life-cycle Manager (ELCM). ELCM manages the testing procedure and allows for automatic execution of experiments. Subsequently, a further architectural block, i.e., the Monitoring and Analytics (M&A) framework [7], is in charge of collecting KPI samples and complementary measurements during the experiment (Monitoring), and process them for a statistical validation of the KPI under test (Analytics).

In this paper, we present the 5GENESIS methodology for KPI validation, which aims to cope with the increased complexity and heterogeneity of testing in the 5G era. The main contributions are:

- we detail the methodology components and corresponding templates, i.e., test cases, scenarios, slices, and ED. The analysis reveals that the methodology can be used by any 5G testbed, even outside the 5GENESIS facility, due to its modular and open-source nature [8];
- we analyze the use of the methodology under the 5GENESIS reference architecture, describing all of the steps leading to a fully automated experiment execution, from the instantiation of needed resources to the collection and analysis of results;
- we showcase the use of the methodology for testing components and configurations in the 5G infrastructure at the University of Málaga (UMA), i.e., one of the 5GENESIS testbeds; and,
- by means of our tests in a real 5G deployment, we provide initial 5G performance assessment and empirical KPI analysis. The results are provided, together with details on adopted scenarios and network configurations, making it possible to reproduce the tests in other testbeds (under the same or different settings) for comparison and benchmarking purposes.

The rest of the article is organized, as follows. Section 2 introduces the background and related work. Section 3 provides an overview of the 5GENESIS reference architecture, with focus on key components that are related to the testing methodology. The details of the testing methodology are described in Section 4. Section 5 describes the realization of the testing methodology in the UMA testbed, including the set up procedures performed and the definition of the related ED. The experimentation results are presented in Section 6. Finally, Section 7 provides the final remarks.

## 2. Background and Related Work

In the following, we provide an overview of background and related work, in order to frame the context and highlight the contribution of this paper. In particular, in Section 2.1, we review the standardization efforts towards the definition of 5G KPIs and corresponding testing and validation methodologies and procedures. In Section 2.2, we summarize recent research activity on 5G testing and field trials, referring to the most relevant 5G experimentation testbeds.

### 2.1. Standardization on 5G KPI Testing and Validation

Playing a key role in the standardization of mobile communications, the 3rd Generation Partnership Project (3GPP) also defines the corresponding tests aiming at verifying that mobile technologies conform to standards. Over the years, 3GPP has defined the testing procedures for Universal Mobile Telecommunications System (UMTS, i.e., 3G), Long Term Evolution (LTE, i.e., 4G) and more recently 5G New Radio (NR).

Focusing on the user-end device, i.e., User Equipment (UE), 3GPP TS 38.521-*X* series details how a 5G NR UE must be verified at radio level, characterizing both transmit and receive parameters in terms of maximum transmit power, receiver sensitivity, and spurious emissions, among others. Conformance to Non-Standalone (NSA) scenarios, which combine NR and LTE cells for the Radio Access Network (RAN), is covered in [9], while NR-only Standalone (SA) is analyzed in [10,11], which address sub-6 GHz and mmWave frequency bands, respectively. Performance aspects that are related to demodulation under different propagation and Signal-to-Noise Ratio (SNR) conditions are defined in [12]. Additional specifications cover the testing of several 5G NR signaling protocols [13,14,15], while [16] focuses on Radio Resource Management (RRM) testing, including the reporting of power and quality measurements, handover latency, timing accuracy, and other functional metrics. Another relevant aspect at the UE side is the battery consumption, for which the Global Certification Forum (GCF) and CTIA-The Wireless Association have proposed a testing methodology in [17], among others.

Moving at the network side, 3GPP TS 28.552 [18] provides specifications for performance measurements of Next-Generation Radio Access Network (NG-RAN), 5G Core (5GC), and network slicing. Moreover, Radio Resource Control (RRC)-related KPIs are measured according to [19,20], while using the measurement template given in [21].

As regards NFV, the European Telecommunication Standard Institute (ETSI) Group Specification (GS) NFV-TST [22] covers the testing of functional blocks of the architecture.

The above activities and documents focus on testing of 5G components separately, and they do not address a fully E2E KPI validation. As analyzed later, our proposed testing methodology allows for both per-component testing and E2E KPIs validation.

On this latter aspect, an initial recommendation is given in 3GPP TR 37.901 [23], which addresses the testing of LTE UE at the application layer. In particular, it specifies the procedure to run throughput tests in a wide set of network scenarios and conditions. When also considering the need for running similar tests for 5G, 3GPP has recently initiated a study on 5G NR UE throughput performance at the application layer, which is progressing as TR 37.901-5 [24], as an evolution of TR 37.901. Our work in 5GENESIS aims at generalizing these recommendations, in order to cover not only throughput, but also other E2E KPIs from specific verticals, e.g., the Mission Critical Push to Talk (MCPTT) access time, for which a test case has been already defined and used [25].

Following the E2E approach, 3GPP TS 28.554 [26] uses a network slicing perspective and currently covers KPIs related to accessibility, integrity, utilization, retainability, mobility, and energy efficiency. These KPIs are based on internal counters of the network, which are only accessible to the network operators; in our methodology, in order to provide direct information on the achievable performance under different 5G use cases, the definitions of KPI privilege instead the end-user perspective, in terms of Quality of Service (QoS) and Quality of Experience (QoE).

TS 28.554 also specifies a template that allows for the categorization of KPIs along with methods, tools, and calculation steps needed for measuring and validating them. However, it lacks specifications for the test sequence and the definition of E2E scenarios. We observe that these aspects are key in order to completely define and take into account the network setup during a test, which includes the relationships between infrastructure, management and orchestration system, measurement probes, user traffic, and so on. As a matter of fact, it is hard to contextualize and compare the obtained results without a proper scenario definition; also, a clear definition of test preconditions is key for running reproducible tests and obtaining reliable results. For this reason, we have defined our testing methodology by taking [26] as a reference, and then extrapolating the provided guidelines towards covering a full list of 5G E2E KPIs. We also aim at a more general and flexible testing approach, tailored on open experimental testbeds. Hence, we propose a test case template that reuses and expands the one in [26] in terms of considered fields, while also addressing clear definitions for test sequence, preconditions, and E2E scenarios.

When considering these aspects, a document by Next Generation Mobile Networks (NGMN) [27] introduces test case sequence and scenarios, with the latter based on the definitions provided in [28]. The goal is the evaluation of 5G NR performance, and the focus is on 3GPP Release 15 (Rel-15), the first step of 5G NR standardization, which primarily considers eMBB and some aspects of URLLC use cases. For both, several KPIs are considered, e.g., latency, user throughput, and mobility, but also capacity, coverage, energy efficiency, and user experience, among others.

The NGMN approach fuses together, in a monolithic definition of test cases, the testing procedures (e.g., test sequence), network configurations (e.g., number of exploited network resources), network conditions (e.g., number of users), and traffic profiles (e.g., used protocol and packet size). As detailed in the next sections, our testing approach is instead modular, i.e., the logical components forming an experiment (test cases, scenarios, and slices) are kept separated, ultimately enabling higher flexibility and adaptability, as well as lightweight extension towards more advanced testing.

Table 1 summarizes the standards and documentation reviewed above.

### 2.2. Research on 5G KPI Testing and Validation

Rigorous testing of 5G E2E solutions is still an emerging research topic, where we find a relevant gap. There are only a few papers that are devoted to this area.

In the context of the TRIANGLE project, the work in [29] proposes a methodology in order to automate the control of all the elements in the E2E 5G network path, while [30] focuses on automating 5G apps. Both of the papers aim to test service level KPIs in a 5G E2E lab setup, while the 5GENESIS methodology described in this paper is tailored for testing and validation in real 5G deployments.

In the context of 5G field trials, several works provide ad-hoc solutions for specific use cases, scenarios, and KPIs. The authors in [31] study throughput for using 15 GHz band. In [32], the authors test throughput and latency in vehicular communications. The work presented in [33] analyses throughput, network energy efficiency, and device connection density. The work in [34] focuses on different methods for generating and evaluating the effect of interference. Moreover, large scale performance measurements on 5G operational networks and off-the-shelf UEs have been recently presented in [35,36]. On the one hand, [35] covers a sub-6 GHz NSA deployment in a dense urban environment, revealing several insights on network coverage, handover mechanisms, UE energy consumption, and E2E throughput, latency, and application performance. On the other hand, [36] discusses throughput, latency, and application performance, along with handover operations, under four different US operators’ networks, with three of them employing NSA deployment with mmWave 5G cells. The above papers provide valuable preliminary insights on 5G achievable performance; however, technology and KPI validation requires dedicated and fully controllable testing environments [3], in order to pinpoint specific causes for the observed performance levels, and ultimately guide towards focused optimized configurations. Our work contributes to these aspects by providing the definitions of E2E KPIs and of testing procedures for reliable performance assessment in dedicated environments.

Several testbeds are being developed for supporting reliable experimentation, testing, and validation of 5G technologies, paradigms, and KPIs, as anticipated in Section 1. Among others, the 5G Test Network (5GTN) presented in [37] integrates cellular access and core networks with SDN/NFV, cloud/edge computing, and Internet of Things (IoT) technologies. Targeting more specific use cases, [38] showcases NFV integration with Unmanned Aerial Vehicles (UAVs), while [39] presents a SDN-based cloud/edge computing platform for IoT management. IoT experimental testbeds for mMTC and low-power devices are also presented in [40,41], while an open solution for E2E network slicing experimentation is given in [42]. Altogether, these testbeds focus on specific 5G/IoT capabilities and use cases, while 5GENESIS aims at combining several technologies and implementing an E2E open facility for the experimentation and testing of heterogeneous verticals. Moreover, the above works do not address the design of procedures for executing properly defined experiments and test cases, which is instead a key 5GENESIS contribution, as detailed in this paper.

5GENESIS works under the 5G-PPP umbrella with other two EU-funded infrastructure projects, i.e., 5G-VINNI (https://www.5g-vinni.eu (accessed on 30 August 2020)) and 5G-EVE (https://www.5g-eve.eu (accessed on 30 August 2020).) Similar goals are also pursued by PAWR testbeds, i.e., POWDER (https://powderwireless.net (accessed on 30 August 2020).), COSMOS (https://cosmos-lab.org (accessed on 30 August 2020)) and AERPAW (https://aerpaw.org (accessed on 30 August 2020)). Altogether, these projects aim at the implementation of distributed 5G facilities in Europe (5G-PPP) and US (PAWR), where researchers, technology providers, stakeholders, and verticals can test their solutions in a reliable and reproducible manner. As analyzed later, the testing methodology that is presented in this paper is currently adopted in the 5GENESIS facility, but it can be easily reused in other 5G testbeds, also thanks to the open-source nature of the software components enabling its application [8].

## 3. Overview of 5GENESIS Approach to KPI Testing and Validation

Before the formalization of the proposed testing methodology, in this section we summarize the 5GENESIS reference architecture and M&A framework, with the latter being a component that is particularly important when the methodology is applied in 5GENESIS testbeds. The methodology has been adopted for experimentation in the 5GENESIS UMA testbed, which is fully compliant with the reference architecture discussed in this section, as detailed in Section 5 and Section 6.

### 3.1. 5GENESIS Reference Architecture

**Figure 1:**
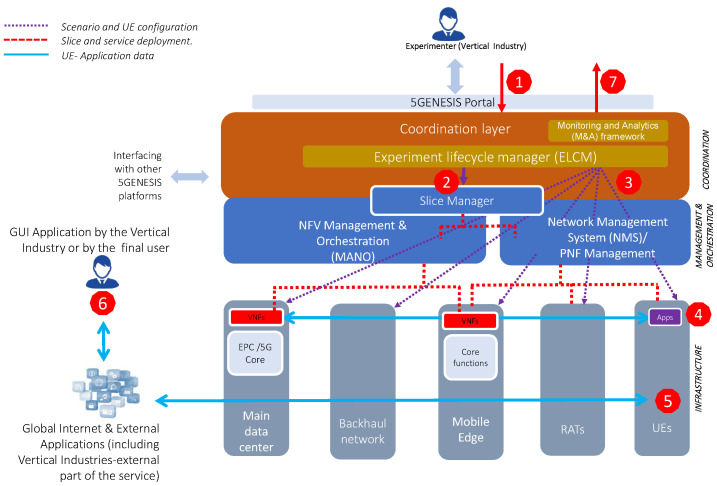
5GENESIS Reference Architecture and Experimentation Flow.

The 5GENESIS approach to the analysis of KPIs for new 5G services developed by vertical industries relies on the construction of experimental platforms with a common architecture, as represented in Figure 1.

The 5GENESIS architecture is structured in three main layers: Coordination, Management and Orchestration (MANO), and Infrastructure Layers. It is an abstraction of the 5G network architecture proposed by 5G-PPP Architecture Working Group in [43], focusing on the experimentation for demonstrating 5G KPIs. Hence, additional components and interactions have been included to introduce an experimentation control plane in the architecture. The 5GENESIS architecture includes the common components for Management and Orchestration (MANO) of services, network slicing, and infrastructure layer. On top of them, the Coordination Layer has been added in order to introduce the experimentation control plane and expose testing and automation features towards the verticals.

Thus, the Coordination Layer includes all of the components related to the control of the experiment execution. Moreover, it also provides the Northbound Interfaces (NBI) towards the 5GENESIS Portal, for sending the description of an experiment and retrieving the results after the execution. More details are provided in [44]. During the experimentation, the ELCM is responsible for the sequencing of experiment lifecycle stages, while maintaining the experiment status and providing feedback on the experiment execution. M&A-related components sitting in the Coordination Layer are responsible for the complete collection and analysis of the heterogeneous monitoring data that are produced during the use of the testbed. In particular, in order to collect the monitoring information from all of the elements of the testbed, the Analytics component retrieves the measurements from a unified database, in which the various measurement probes ingest, either in real-time or at the end of each experiment session, the measurements for long-term storage and data post-processing.

In the MANO Layer, the Slice Manager is in charge of the configuration and deployment of the slices. For doing this, it relies on the Network Function Virtualization Management and Orchestration (NFV MANO), which is responsible for the orchestration and lifecycle management of Network Services and Virtual Network Functions (VNFs), and on the Network Management System (NMS), which is in charge of the management of Physical Network Functions (PNFs).

The Infrastructure Layer implements the E2E 5G network, including UEs, RAN, core network, main data center, and mobile edge. The main data center and mobile edge refer to the Network Function Virtualization Infrastructure (NFVI) located at the core and at the edge of the network, respectively. Section 4 provides more details on how to relate this architecture with the experimentation methodology and how a sample experiment is run.

### 3.2. 5GENESIS M&A Framework

The KPI validation is managed by the M&A framework [7], which targets the collection of KPIs and complementary measurements, aiming at reliably validating the former while exploiting the latter in order to verify the infrastructure status during the experiment. Integrated onto the 5GENESIS architecture, the M&A framework currently includes several Monitoring tools and both statistical and Machine Learning (ML) Analytics functionalities. It is formed by three functional blocks:

- Infrastructure Monitoring (IM), which collects data on the status of architectural components, e.g., UE, RAN, core, and transport systems, as well as computing and storage distributed units;
- Performance Monitoring (PM), which executes measurements via dedicated probes for collecting E2E QoS/QoE indicators, e.g., throughput, latency, and vertical-specific KPIs;
- Storage and Analytics, which enable efficient data storage and perform KPI statistical validation and ML analyses.

The KPI statistical validation plays a key role in the 5GENESIS methodology. As detailed in Section 4, 5GENESIS adopts a per-iteration validation approach. Hence, an experiment, as described in the corresponding ED, consists of executing a given number of measurement iterations. The validation is first performed on each iteration, and then on the entire experiment. The procedures for calculating the statistical indicators that validate the KPIs are specified in the test case template, which is part of the ED along with scenario and slice configurations.

## 4. Formalization of the Experimentation Methodology

The proposed methodology has been composed in order to specify the actions that are needed for the execution of an experiment on 5G infrastructures; and, also, to structure the information flow required from the design and set of an experiment to the collection of the results. When considering the intense interest from vertical industries to use and assess the benefits of 5G, the proposed methodology could be used as a basis for any experimentation process in 5G infrastructures and, as such, it moves beyond its specific application in the infrastructure used in this paper. The open source notion of the major components that realise the methodology (referring mainly to the Open5GENESIS suite [8]) contributes a lot towards that direction.This section provides the specifications for each of the key concepts of the 5GENESIS experimentation methodology. More precisely, we focus on the formalization of the experimentation methodology. i.e., the process of i) specifying templates for the three key entities that are mentioned above, namely, the test cases, the scenarios, and the slices, and ii) identifying all of the additional to those templates information needed to run the experiment. The test cases, the scenarios, and the slices, as well as the complementary information, are needed in order to fully self-define an experiment.

### 4.1. Test Case

A test case describes the targeted KPI and the instructions for configuring the network and performing the measurement(s) to compute it.

Table 2 reports an example of a test case that is devoted to measure the maximum downlink (DL) throughput at the application level. User Datagram Protocol (UDP) traffic has been used because it is more appropriate for this assessment, in comparison to Transmission Control Protocol (TCP), where the measurements could be affected by flow and congestion control mechanisms. Additional test cases can be found at [25].

The test case specifies the end points where the measurements are collected, the measurement tools and corresponding configurations, how to calculate the throughput, the sequence of actions for executing the test case, and the complementary measurements that can be used for troubleshooting and understanding the obtained values. More precisely, the following information is provided in the test case template:

**Target KPI**: this field includes the definition of the target KPI. Each test case targets only one KPI (main KPI). However, secondary measurements from complementary KPIs can also be added (see “Complementary measurements” field of this template). The definition of the main KPI specifies the related target metric, the ID of which is declared in the first row of the template. More precisely, the definition of the main KPI declares at least the reference points from which the measurement(s) are performed, the underlying system, the reference protocol stack level, and so on. Within the 5GENESIS experimentation methodology, the term “metric” refers to a generic high-level definition of a target quality factor (attribute) to be evaluated, i.e., a definition that is independent of the underlying system, the reference protocol layer, or the tool used for the measurement. The initial set of identified metrics is specified in [25], based on an abstraction of the set of 5G KPIs defined by 5G-PPP in [45].

**Methodology**: this field declares the procedure and configurations relevant for defining the experiment execution, including the acceptable values for the experiment duration, the iterations required to obtain statistically significant results, and the measurement interval, to mention a few.

**Calculation process and output**: this field describes the processing performed on the KPI samples that allows to derive the statistical indicators for the executed test case, ultimately enabling the KPI validation. As reported in the “Methodology” field, the experiment should be executed for a statistically significant number of iterations, e.g., *I*, in order to obtain an accurate picture of the KPI. An example of calculation process and corresponding output is given in the test case template that is presented in Table 2.

**Complementary measurements**: this field defines a list of secondary parameters useful to interpret the values of the target KPI. On the one hand, getting these measurements is not mandatory for the test case itself; on the other hand, the M&A framework can exploit them in order to provide more insights on the experiment execution. As a first step, an overview of the complementary measurements (e.g., in terms of per-iteration and test case statistical indicators) can be provided to the experimenter along with the results that are related to the target KPI. Moreover, such measurements can help to pinpoint anomalous and unexpected behaviors, provide correlation and predictive analyses via ML tools, and trigger architectural improvements for next experiment executions.

**Preconditions**: a list of test-specific information about equipment configuration and traffic description is provided in this field. Additionally, the description of the initial state of the System Under Test (SUT), required to start executing a test case sequence, is provided.

**Applicability**: this field reports a list of features and capabilities which are required by the system in order to guarantee the feasibility of the test.

**Test case sequence**: this field specifies the sequence of actions to be performed during the execution of the test case.

Besides the test case reported in Table 2 above, the 5GENESIS Consortium has already defined several test cases, including the ones for assessing latency KPIs (see Section 5 and Section 6). Test cases covering further 5G KPIs are also available in [25].

### 4.2. Scenario

The scenario template includes information that is related to network and environment configurations, and is related to the technologies supported in the experimentation platform. From the performance perspective, the scenario quantifies the parameters that affect the values of the KPIs to be measured.

The parameters that are part of the scenario definition are different from those that are specified for the slice. The scenario parameters establish the working point of the network, including UE location and mobility conditions, and they provide a guideline for the definition of network conditions, in order to reproduce realistic situations where to perform the experiments. Hence, the scenario definition is dependent on the infrastructure. Table 3 provides an example of the description of a 5G NR NSA scenario. The accessible parameters in each testbed may differ, depending on the type of equipment available in each of them. Hence, the scenario template aims to be a guideline for the key parameters that must be known to gain a clear understanding of the results that were obtained during the testing and identify the context in which the validation is performed.

### 4.3. Slice

Network slicing support is part of the new Service-Based Architecture (SBA) specified by 3GPP in TS 23.501 [46]. A network slice is defined as “a complete logical network (providing Telecommunication Services and Network Capabilities) including Access Network (AN) and Core Network (CN)”. The management and orchestration of network slices is specified in 3GPP TR 28.801 [47]. The list of Standard Slice Types (SST) specified in [46] includes three slices: *eMBB slice*, suitable for 5G eMBB services; *URLCC slice*, oriented towards 5G URLLC use cases; and, *MIoT slice*, for massive IoT and mMTC applications. However, the standard also contemplates the use of non-standard slices.

In the 5GENESIS methodology, the slice specifies the E2E resources specifically allocated in the network in order to fulfill the performance requirements of the solution under test. A full description of the slicing mechanisms supported in 5GENESIS can be found in [48] and in the corresponding GitHub repository [49]. The template that is used to define the specific slice configuration is based on the Generic Network Slice Template (GST) defined by the Global System for Mobile Communications Association (GSMA) in [50], and Listing 1 shows a GST example. Hence, a testbed defines different instances of the slice template, one per supported slice. Subsequently, the slice is configured, depending on the capabilities of the infrastructure layer, which is, the mapping between the parameters defined in the template and the values configured in each network component depends on the available equipment, which can range from commercial base stations to emulators or software-defined solutions. However, the mapping is transparent to experimenters and vertical use case owners, which just need to choose among the list of slices offered by the testbed. The selection that is made by the experimenter is finally used to fill in the slice ID field in the ED.

### 4.4. Experiment Descriptor

The ED is a data structure that includes all of the values that are required for completely defining an experiment execution, as shown in Listing 2. It includes the following fields:

Experiment type: it is part of the definition of the experiment. The 5GENESIS experimentation methodology currently supports three types of experiments. However, the methodology is sustainable and can be easily extended with new types of experiments. The standard experiments are based on the test cases specified by the 5GENESIS Consortium, and enable the comparison and benchmarking of different variants of a same solution (devices, services, applications, etc.). The custom experiments are those that are defined based on specific requirements of the solution under test. For example, in a custom test case, the measurements could be specific with respect to a product being tested. Finally, MONROE experiments are containerized experiments initially designed in the MONROE project [51,52]. Within 5GENESIS, a virtual MONROE node has been developed that decouples the MONROE software from the MONROE hardware infrastructure. The MONROE virtual node can be used at the UE, providing a generalized mechanism for running arbitrary containerized experiments without any need to update the experimentation framework.

**Automated**: it indicates if the experiment is fully automated, i.e., no human intervention is required for the execution. Automated experiments target the execution of exhaustive testing looking for the benchmarking of the solutions under test. Human intervention is expected when the network can be configured automatically but the applications and UEs are operated by the vertical use case owners.

**Test Cases**: it includes the IDs of the test cases selected to be executed in the experiment.

**UEs**: it includes the ID(s) of the UE(s) used during the experiment.

**Slices**: it contains the ID(s) of the slice(s) to be used during the experiment. The full slice definition is available in a separate data structure, as described in Section 4.3.

**NSs**: it refers to the Network Services (NSs) that are used during the experiment. Depending on the target of the experiment, the NSs could be deployed at different stages of the experiment. This stage is specified in the test case.

**Scenarios**: the ID(s) of the scenario(s) to be used during the experiment are listed in this field. The full scenario definition is available in a separate data structure, as described in Section 4.2.

**Exclusive Execution**: it controls the scheduling of different experiments in the testbed. An Exclusive experiment is not executed at the same time with other experiments.

**Reservation Time**: it defines the duration of the experiment when automation is not enabled.

**Application**: this field depends on the type of experiment and test case. It may be used for standard and custom experiments to specify the application to run (e.g., in the second case, the application may be directly defined by the experimenter). In the case of MONROE experiments, the field defines the container to deploy in the MONROE virtual node.

**Parameters**: this field depends on the type of experiment. In particular, it is used for specifying customized parameters in custom experiments, but it could be also used to include tool-specific settings not explicitly reported in the test case of a standard experiment. For MONROE experiments, the field provides the configuration for the containers involved in the experiment.

**Remote**: this field is necessary to support the execution of distributed experiments, possibly involving two testbeds. It is used to identify the secondary testbed that is part of the experiment.

**Remote Descriptor**: it contains a secondary ED with the values that are required to configure the experiment execution in the remote testbed.

**Version**: it specifies the ED version in use, so that the testbed can customize the handling of the ED according to any future modification, while keeping compatibility with older EDs.

**Extra**: this field can be used to add further information. For example, it can be useful for adding debugging or tracing information, or to support extra functionalities without changing the ED format.

Each testbed needs to create a registry detailing the type of experiments, and the list of supported test cases, scenarios, slices, NSs, and UEs, since this information is required to fill in the ED. As part of the implementation open-sourced in [8], 5GENESIS makes available a Web Portal, where this information can be easily configured and the verticals just need to select between the options available in the testbed to define an experiment.

### 4.5. Experiment Execution

The experiment workflow is depicted in Figure 1, on top of the 5GENESIS reference architecture. In terms of execution flow, labels 1 to 7 represent the steps for describing an experiment (1), to execute it (2 to 6) and to report results (7). Next, we illustrate the execution of an experiment with an example.

Assume that a service provider would like to assess the delay perceived at the application level in a certain device. First, the experiment is configured via the Portal. The chosen parameters will be used in order to fill in the fields of the ED. In the example, the experiment requests the execution of a Round Trip Time (RTT) test in a static scenario with LoS and good coverage, and the usage of an URLLC slice. Subsequently, the ED is delivered to the Coordination Layer first and MANO Layer then, where the Slice Manager deploys and configures the entire slice indicated by the experimenter in the ED. In the third step, the scenario is configured via the configurations that are specified in the corresponding scenario template selected by the experimenter. Among other, in this specific example, a ping application is installed in the device under test. The scenario can potentially affect the radio access technology (e.g., the signal strength of the base station), as well as the backhaul and core networks (e.g., the amount of background traffic in the backhaul). In the fourth step, the ELCM initiates the ping client, as well as the monitoring probes collecting all of the complementary measurements specified in the test case (or at least the ones available). The activation of the ping client triggers the 3GPP signaling in order to establish the data connection. In this example, the experiment test case defines a ping between the UE and the Packet Gateway (P-GW) located at the core network, so we can skip steps five and six in the execution flow, devoted to the testing of services deployed outside of the testbed (e.g., at the vertical facility). Finally, M&A post-processes the measurements and reports the obtained KPI statistical indicators to the experimenter.

The source code for the components described in this section (Portal, ELCM, Slice Manager, etc) is available under Apache license 2.0 in [8]. The license allows modifying and using the software without restrictions. The main purpose of this open initiative is to provide a general testing framework for 5G infrastructures. The framework is flexible enough to cover the testing requirements of 5G verticals; moreover, it enables the comparability and reproducibility of experiments and results across 5G infrastructures having similar features.

The repository in [8] also includes the probes that were used in Android devices to collect the radio information, the iPerf and ping agents for Windows PCs and Android devices, and a streaming agent for video streaming tests. A manual for installing and using all of the components is also available [44].

## 5. Experimental Setup

This section and the following one present a practical application of the testing methodology in the 5G deployment located at the UMA campus, which is described in Section 5.1. The methodology is applied in order to quantify throughput and latency KPIs in different networks scenarios. We define the test cases in Section 5.2, while presenting the scenarios and used slice in Section 5.3 and Section 5.4, respectively. Finally, we report the EDs for three experiments executed for our validation purposes in Section 5.5.

### 5.1. 5G Deployment at UMA Campus

This section describes the 5G deployment at UMA campus, where the experiments have been executed. 3GPP TS 37.340 [53] considers different options for the deployment of 5G networks. The options range from purely 5G solutions, deployed independently from the existing network, to hybrid solutions, which combine part of the existing infrastructure with 5G NR.

Becasue of the high costs of building native 5G solutions, most operators currently prefer to lean on and evolve from the existing LTE infrastructure [35,36]. In particular, the combination of 4G and 5G radio access connected to an LTE EPC is known as NSA operation Option 3x architecture. This option is also called eUTRA New Radio-Dual Connectivity (EN-DC). Dual Connectivity (DC) was introduced in 3GPP in order to allow a UE to simultaneously transmit and receive data on multiple component carriers from/to two cell groups via a master eNB (MeNB) and a secondary eNB (SeNB). In Option 3x, DC is used in order to allow a device to connect to both 4G and 5G NR.

**Figure 2:**
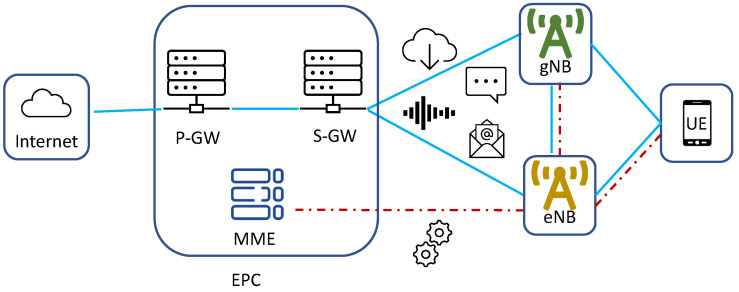
Core and Radio Access Network (RAN) configurations as per 5G NR NSA Option 3x.

For this reason, the initial UMA pilot also follows the NSA Option 3x architecture. In Option 3x, the UE is connected to an eNB that acts as MeNB, and to a gNB that acts as SeNB, as shown in Figure 2. As said, there is no 5GC in this option and the gNB does not connect to the Mobility Management Entity (MME) in the EPC. The gNB connects to the eNB to receive requests to activate 5G bearers via X2 interface. Data bearers can be handled by the MeNB or SeNB, or split between these two. For the measurements that are discussed in this paper, the data are handled by the SeNB only (the gNB), which is the most popular Option 3x variant.

**Figure 3:**
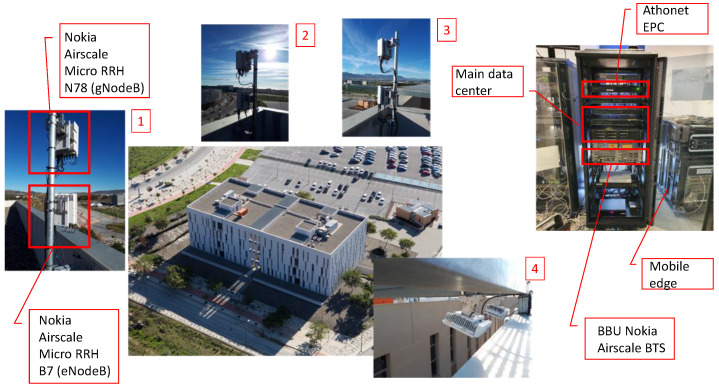
5G NR NSA deployment at UMA campus. The operation band is also reported for the Radio Remote Heads (RRHs) forming gNBs/eNBs, numbered from (1) to (4).

Figure 3 shows the four paired gNBs/eNBs currently deployed in the UMA campus, along with other infrastructure components, i.e., the server rack hosting the EPC from Athonet, the Baseband Unit (BBU) from Nokia, and the main data center, in which OpenStack has been adopted as the Virtual Infrastructure Manager (VIM) and Open Source MANO (OSM) as the solution for NFV management and orchestration. Moreover, the mobile edge solution provided by Telefonica is based on OpenNebula and OSM. More details regarding the testbed components can be also found at [54].

### 5.2. Test Case Definition

We have defined two test cases for throughput and latency KPI validation, referred to as TC_THR_UDP and TC_RTT, respectively.

The details of the TC_THR_UDP test case are provided in Table 2, while TC_RTT is a ping-based latency test. Similarly to TC_THR_UDP, TC_RTT also specifies 25 iterations; each iteration lasts two minutes, during which Internet Control Message Protocol (ICMP) ping is performed with ICMP ECHO REQUEST packets of 56 bytes, sent at a rate of 2 Hz. The ping source is an application running in the UE and the destination is the P-GW of the core network. The calculation process and output is the same as defined for TC_THR_UDP in Table 2.

### 5.3. Scenario Definition

Three different scenarios have been defined. Table 3 shows the SC_LoS_PS scenario, which refers to a LoS situation, where Proactive Scheduling (PS)—a vendor-specific feature in the gNB—is activated. The other two scenarios, which are denoted SC_LoS and SC_NLoS_PS, are similar to SC_LoS_PS, but differ in PS deactivation (SC_LoS) or UE location, which is non LoS (NLoS) in SC_NLoS_PS. (For simplicity, in the following we will also refer to SC_LoS_PS (and SC_LoS) and SC_NLoS_PS as LoS and NLoS scenarios, respectively, with clear mapping between the two notations.) Next, we discuss, with more detail, the configurations in Table 3.

After identifying the technology (5G NR) and the deployment mode (NSA), the next scenario parameter is the LTE to NR frame shift. This setting governs the relative time difference between the start of the frame timing for both technologies. With a shift of 3 ms, subframe 0 in one technology will start together with subframe 3 in the other.

Regarding the cell power, the 5G NR cell transmits at 10 W (40 dBm), with an average power density of 0.25 W/MHz in the full channel, which is located in n78 band, i.e., 3300 to 3800 MHz.

A 5G NR channel of 40 MHz is nominally adopted, with 5G NR numerology 1 and SubCarrier Spacing (SCS) of 30 kHz. In this case, the maximum number of Physical Resource Blocks (PRB) that can be used is 106, as per 3GPP TS 38.101-3 ([55] Section 5.3.2). Moreover, the 5G carrier is currently configured to use two antennas. Hence, we can enable a maximum of two layers to increase the throughput, taking advantage of the Multiple Input Multiple Output (MIMO) diversity using close loop spatial multiplexing. We also use a single beam and a maximum modulation of 256-Quadrature Amplitude Modulation (256-QAM). As per 5G NR Modulation and Coding Scheme (MCS) mapping in TS 38.214 ([56] Table 5.1.3.1), 256-QAM modulation is enabled with the maximum MCS of 27, 8 bits encoded per OFDM symbol, and a coding rate of 948/1024. In this setup, the L1 payload spectral efficiency results of 7.4063 bits per symbol.

The use of Time Division Duplex (TDD) mode, together with 30 kHz SCS, results in a time resolution of 500 μ s for each individual transmission. Within such a time slot, there are 14 OFDM symbols. Of those, 11 symbols are effectively used for data transmission, as the other three are used for control information and demodulation of reference signals. At frame level, a 2/8 pattern translates to DDDDDDDSUU, i.e., a 5 ms pattern of seven DL slots (two patterns per 10 ms frame), one special slot, and two uplink (UL) slots is used. When considering that our test cases focus on DL throughput, only the DL slots are effectively used for user data transmission.

With the above setup, the maximum theoretical DL throughput is of approximately 290 Mbps, derived from multiplying the number of layers (2), PRBs (106), OFDM data symbols (11), and bits per symbol (8 bits), with the coding rate (0.926), the number of slots in DL used for data transmission (7), the patterns per frame (2), and the number of frames in one second (100).

As anticipated above, we use PS in SC_LoS_PS and SC_NLoS_PS scenarios, which is a feature that is available in the gNBs of the UMA deployment. On the one hand, in a generic scheduling configuration, when a UE needs to transmit data after being inactive for a long interval, it has to request an UL grant via scheduling requests or even a dedicated connection through Random Access Channel (RACH). Once the RACH contention is resolved or a scheduling request is successfully received, the UE will be able to transmit data. This process takes non negligible time and it may not be optimal for delay-critical use cases. On the other hand, when PS is used, the transmission latency is minimized, since the gNB pre-assigns resources to the UE, even if the latter has not explicitly requested them yet. This may clearly bring some overhead, but it could be an optimal solution for scenarios employing limited amount of mobile devices, where the delay is the critical aspect, e.g., a private 5G network dedicated to time-sensitive communications. Specifically, in our test, we adopt a PS configuration that allocates UL grants to the UE in every UL suitable opportunity, which, in this specific configuration, was 10% of the total TDD slots.

Finally, we further comment on the difference between LoS and NLoS scenarios, which clearly resides on the higher propagation losses in the latter case. In NLoS, the used gNB is not directly oriented towards the device under test, and the signal is obstructed by external and internal building walls. We executed preliminary measurements in order to characterize the additional attenuation of NLoS as compared to LoS, which resulted in about 60 dB lower received signal level and 11 dB worse signal to noise ratio when compared to the LoS case. In both cases, the UE was static and there was no background traffic in the system.

### 5.4. Slice Definition

The configurations for the slice used in our experiments are reported in Listing 1. The template defines the achievable DL/UL throughput for the whole slice, the guaranteed DL/UL throughput supported by the slice per UE, and the Maximum Transfer Unit (MTU). This is a default slice, in which all of the radio resources are available for the UE.

### 5.5. ED Definition

In this section, we show the EDs for three experiments, which have been executed in the UMA 5G deployment while using the methodology presented above. Section 6 analyzes the obtained results.

We have defined a first experiment, named Experiment 1, aiming at quantifying throughput and latency KPIs in our deployment under LoS and NLoS scenarios. Listing 3 shows the ED for this experiment. In 5GENESIS terms, Experiment 1 is a standard experiment, which sequentially executes two standard test cases, i.e., TC_THR_UDP and TC_RTT, in SC_LoS_PS and SC_NLoS_PS scenarios, by using the default available slice and a single 5G NR device, denoted UE_1. Because the applications are already defined in the test cases and no NSs are used, the Application and NSs fields of the ED can be omitted.

Because Experiment 1 is executed with PS activated, we have designed Experiment 2 aiming at quantifying the impact of such a scheduling configuration on the observed latency. Along with the difference between LoS and NLoS (Experiment 1), Experiment 2 represents another example highlighting the possible impact of changing a scenario configuration on the validation of a KPI.

Experiment 2 is also a 5GENESIS standard experiment based on a standard test case, as shown in Listing 4. In particular, we focus on TC_RTT test case, and run it in a single device (UE_1), in both SC_LoS_PS and SC_LoS scenarios. This ultimately allows for isolating the effect of PS.

Finally, we define a third experiment, i.e., Experiment 3, to showcase the flexibility of the proposed methodology. In particular, Experiment 3 highlights how the methodology can be used for benchmarking purposes, and for underlining peculiar behaviors in off-the-shelf devices, requiring further inspection; hence, as reported in Listing 5, we execute the TC_THR_UDP test case in the SC_LoS_PS scenario, while using UE_1 and a second 5G NR device from a different vendor, denoted UE_2.

## 6. Experimental Results

In this section, we discuss the results that were obtained by executing the experiments defined in Section 5.

### 6.1. KPI Nalidation across Different Scenarios

We start with Experiment 1, which quantifies throughput and latency KPIs in LoS vs. NLoS scenarios (and PS activated), and it provides an overview of the full usage of the proposed methodology.

Table 4 and Table 5 report several statistical indicators (and corresponding 95% confidence intervals) for TC_THR_UDP and TC_RTT test cases in both scenarios, respectively. Along with the main KPIs, i.e., UDP throughput for TC_THR_UDP and RTT for TC_RTT, both tables also report an overview of selected complementary measurements, in terms of the average values measured over the entire test case duration.

As regards TC_THR_UDP, we observe that the UDP throughput in the LoS scenario is stable at around 180 Mbps, and significantly drops in NLoS, where it achieves an average value of 15 Mbps. Focusing on LoS scenario, we see that such a throughput is rather far from the theoretical maximum calculated in Section 5.3. Because our monitoring probes also allow for the collection of throughput samples at different protocol stack layers and, in particular, at Packet Data Convergence Protocol (PDCP), we investigate this aspect more in detail and report the results in Experiment 3, where we also compare the UE_1 and UE_2 performance (cf. Section 6.3).

When considering TC_RTT, we observe an average RTT of about 12 ms in the LoS scenario, which doubles to 26 ms in the NLoS scenario. In the second scenario, we also see larger variance in the KPI samples compared to the first scenario, which instead appears extremely stable. In particular, an extremely high value for the maximum RTT is registered (about 555 ms); this is followed by a large confidence interval and it significantly differs from the 95% percentile indicator (about 48 ms), leading to the conclusion that such a value may be a unique deviation not completely representative of the performance achievable in the scenario under analysis.

**Figure 4:**
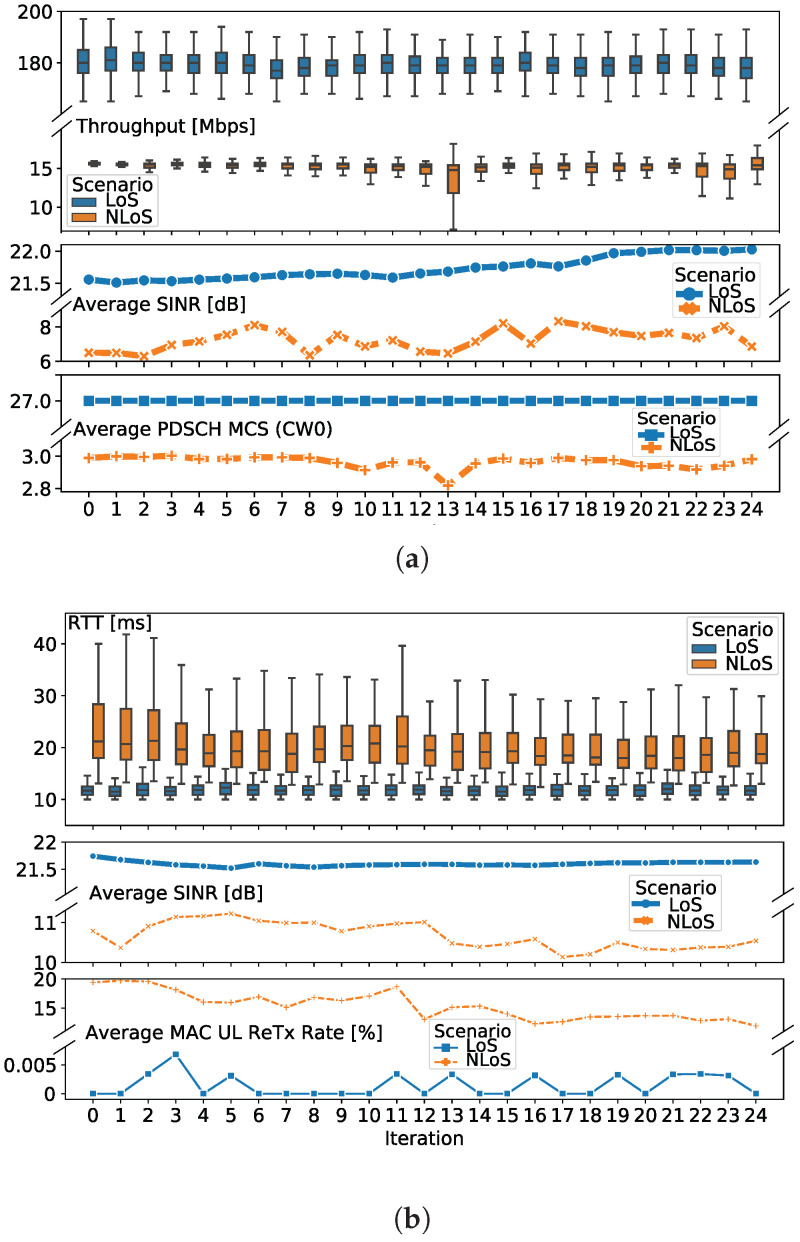
Experiment 1: Per-iteration statistics in SC_LoS_PS and SC_NLoS_PS scenarios and UE_1. UDP Throughput (**top**), average SINR (**middle**) and average PDSCH MCS CW0 (**bottom**) are reported for TC_THR_UDP test case (**a**). RTT (**top**), average SINR (**middle**), and average MAC UL ReTx Rate (**bottom**) are reported for TC_RTT test case (**b**).

The proposed methodology defines such indicators as a final statistical agglomeration of the measurements collected over several iterations (25 in our test cases), as defined in the “Calculation process and output” field of Table 2. As such, the methodology also allows for a more detailed, per-iteration overview of the same measurements. To showcase this aspect, we report, in Figure 4, the KPI statistics per iteration (in a boxplot format), for LoS/NLoS scenarios and TC_THR_UDP (Figure 4a) and TC_RTT (Figure 4b) test cases. Selected complementary measurements are also depicted for both test cases.

One the one hand, Figure 4a depicts the average SINR and the MCS index, which help to understand the context in which the measurements were taken. Among others, in the NLoS scenario, low SINR values directly map to low MCS index and, thus, the resulting low throughput. Given the poor signal level in NLoS, the scheduler reduces the amount of transmitted data to increase the redundancy and maintain the error rate at a reasonable level. As a matter of fact, the BLER observed in the NLoS scenario is lower than 10%, which is a typical target value when the propagation conditions are not favourable enough, so that the Hybrid Automatic Repeat reQuest (HARQ) mechanism can operate optimally. However, given that the MCS index is reduced to 3, the obtained throughput is about 15 Mbps, extremely lower than the throughput of about 180 Mbps obtained in the LoS case, where MCS index is the maximum (27) across all iterations, leading to the use of a high modulation order (256-QAM).

On the other hand, Figure 4b shows that the RTT increase in the NLoS scenario directly maps with degraded radio conditions, particularly in terms of SINR, which, indeed, require a higher retransmission rate when compared to nearly-zero retransmissions observed in LoS conditions.

### 6.2. KPI Validation across Different Configurations

We now move on to Experiment 2, which aims at highlighting the sole impact of PS on the achievable RTT. Hence, as compared to Experiment 1, this experiment focuses on the TC_RTT test case and runs it in LoS scenarios with vs. without PS activation.

**Figure 5:**
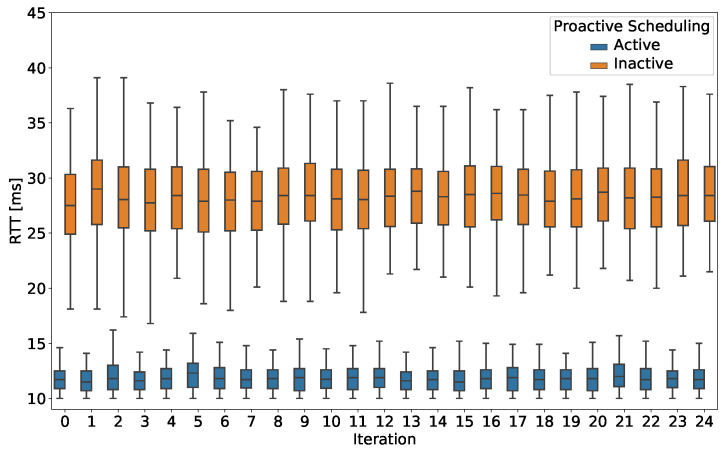
Experiment 2: RTT per-iteration statistics for TC_RTT test case, in SC_LoS_PS (PS active) and SC_LoS (PS inactive) scenarios and using UE_1.

Figure 5 shows the per-iteration statistics of RTT, for TC_RTT test case in SC_LoS_PS and SC_LoS scenarios when using UE_1. The comparison shows a significant impact of PS on the average RTT as well as on its variance. When PS is not active, the observed RTT ranges between 20 and 40 ms, with most of the results being concentrated between 25 and 30 ms. When PS is active, there is a smaller variance, with most of the values concentrated around 11 and 13 ms, and the remaining values in the interval from 10 to 15 ms. The result ultimately validates the impact of PS and its potential suitability for time-critical low-dense use cases, possibly delivered via a dedicated 5G network.

### 6.3. KPI Validation across Different Technologies

The goal of Experiment 3 is to benchmark different UEs by means of their nominal achievable throughput, while also deepening the inspection of the mechanisms across layers of the 5G NR protocol stack. To this aim, it embeds the TC_THR_UDP test case and LoS scenario, and it is alternatively executed on UE_1 and UE_2, two 5G NR devices that are provided by different vendors. Finally, its focus is on comparing UDP and PDCP throughput statistics.

**Figure 6:**
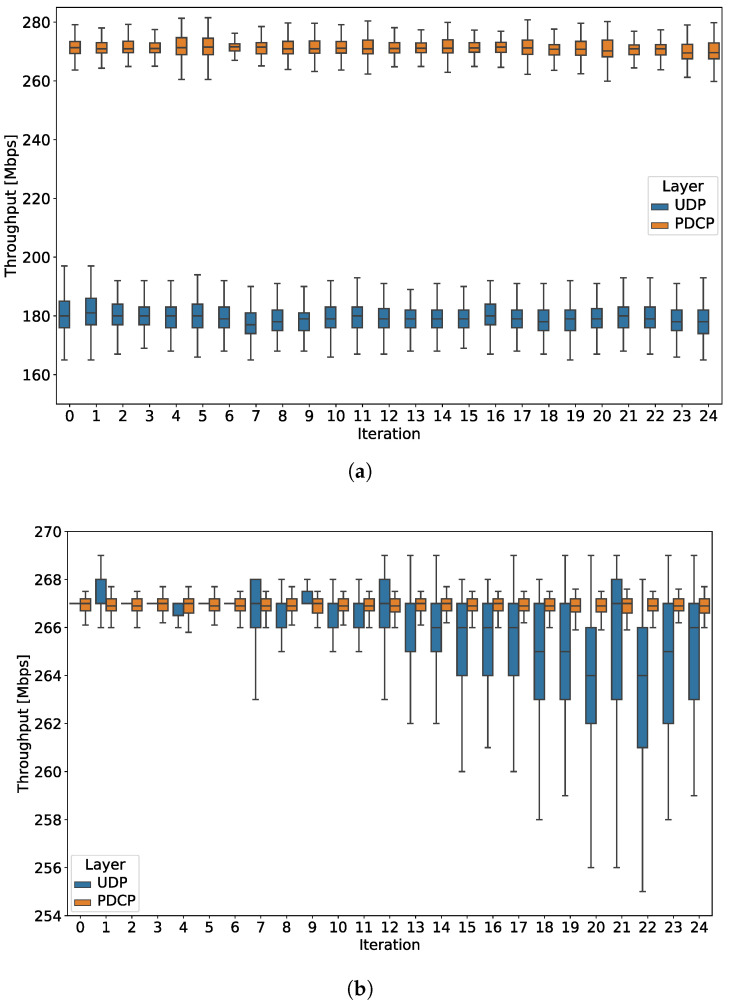
Experiment 3: UDP and PDCP Throughput per-iteration statistics for TC_THR_UDP test case and SC_LoS_PS scenario. Results for UE_1 and UE_2 are reported in (**a**,**b**), respectively.

Figure 6a reports per-iteration statistics of UDP and PDCP throughput for both devices. As already analyzed in Experiment 1, we observe a UDP throughput of about 180 Mbps for UE_1 (Figure 6a). Interestingly, we see instead a PDCP throughput of around 271 Mbps, close to the theoretical maximum calculated in Section 5.3. Hence, under higher data rates possibly sustained by good coverage (i.e., LoS), UE_1 shows a quite constant and rather significant throughput drop, while moving at the application level. When considering UE_2 (Figure 6a), we see that the PDCP throughput stabilizes at a slightly lower value compared to UE_1 (around 267 Mbps). In parallel, the UDP throughput is close to its PDCP counterpart at the experiment kick-off, and then it shows a decreasing trend over the experiment duration, resulting in a difference up to 10 Mbps for the extreme values in the final iterations. These are both unexpected behaviors, repeatedly detected on the devices under test, which call for further inspection and analysis. As highlighted by these results, the statistical analysis that is currently supported in the methodology enables quantifying KPI trends, validating nominal values, and detecting possible malfunctions and anomalies leading to performance issues. We are currently working on embedding further ML-based analytical functionalities in the methodology, which could provide more insights while potentially pinpointing the root cause of the observed performance. This would ultimately lead to the introduction of enhanced schemes for improving cross-layer configurations and performance across the 5G system.

Figure 6a reports per-iteration statistics of UDP and PDCP throughput for both devices. As already analyzed in Experiment 1, we observe a UDP throughput of about 180 Mbps for UE_1 (Figure 6a). Interestingly, we see instead a PDCP throughput of around 271 Mbps, close to the theoretical maximum calculated in Section 5.3. Hence, under higher data rates possibly sustained by good coverage (i.e., LoS), UE_1 shows a quite constant and rather significant throughput drop while moving at application level. When considering UE_2 (Figure 6a), we see that the PDCP throughput stabilizes at a slightly lower value when compared to UE_1 (around 267 Mbps). In parallel, the UDP throughput is close to its PDCP counterpart at the experiment kick-off, and then it shows a decreasing trend over the experiment duration, which results in a difference up to 10 Mbps for the extreme values in the final iterations. These are both unexpected behaviors, repeatedly detected on the devices under test, which call for further inspection and analysis. The statistical analysis currently supported in the methodology enables quantifying KPI trends, validating nominal values, and detecting possible malfunctions and anomalies leading to performance issues, as highlighted by these results. We are currently working on embedding further ML-based analytical functionalities in the methodology, which could provide more insights while potentially pinpointing the root cause of the observed performance. This would ultimately lead to the introduction of enhanced schemes for improving cross-layer configurations and performance across the 5G system.

## 7. Conclusions

In this paper, an open and flexible experimentation methodology for 5G KPI validation is introduced. This methodology overcomes the need for an end-to-end systematic experimentation methodology that allows properly defined and repeatable experiments. Such a methodology brings a key step towards a reliable and fair benchmarking of 5G services and applications.

The methodology has been applied in order to calculate the maximum achievable throughput and the latency in 5G NR scenarios. In particular, three different scenarios have been designed and the ability of the methodology has been demonstrated to (a) validate different KPIs under heterogeneous network scenarios, (b) quantify the impact of specific network configurations on the performance, and (c) pinpoint issues in off-the-shelf 5G devices.

Additionally, one of the designed scenarios has enabled to reach a minimum latency of 10 ms via activation of proactive scheduling mechanisms. This gives key insights on how to reach low latency in 5G deployments based on Release 15, which is focused on eMBB use cases. For URLLC applications, such as mission critical communications or edge computing scenarios, proactive scheduling can be a key parameter.

In future work, we will investigate testing requirements of 5G verticals such as industrial use case. We will focus on identifying specific KPIs and formalize their measurement and calculation procedures, while providing the means for executing the corresponding experiments. Moreover, we are also upgrading the 5G infrastructure to support 5G NR SA. The methodology will then be applied to check the relative performance with respect to NSA.

## Figures and Tables

**Table 1:** Summary of standards for 5G testing and Key Performance Indicator (KPI) validation.

Main Component	Reference	Focus
	3GPP TS 38.521-3 [9]	5GNR UE RF conformance. NSA
	3GPP TS 38.521-1/2 [10,11]	5GNR UE RF conformance. SA FR1/FR2
UE	3GPP TS 38.521-4 [12]	5GNR UE RF conformance. Performance
	3GPP TS 38.523-1/2/3 [13,14,15]	5GNR UE protocol conformance
	3GPP TS 38.533 [16]	5GNR UE RRM conformance
	GCF/CTIA [17]	Battery consumption
RAN/Core	3GPP TS 28.552 [18]	RAN, 5GC, and network slicing performance
RAN	3GPP TS 32.425 [19], TS 32.451 [20]	RRC aspects
NFV	ETSI GS NFV-TST 010 [22]	Testing of ETSI NFV blocks
	3GPP TR 37.901 [23], TR 37.901-5 [24]	4G/5G throughput testing at application layer
E2E	3GPP TR 28.554 [26]	Slicing performance at network side
	NGMN [27]	eMBB and uRLLC (partial) KPI testing
Network elements	3GPP TS 32.404 [21]	Measurement templates

**Table 2:** Test case for assessing the maximum throughput available at the application level.

Test Case: TC_THR_UDP		Metric: Throughput
**Target KPI**: UDP Throughput The UDP Throughput test case aims at assessing the maximum throughput achievable between a source and a destination. Source of packets: Measurement probe acting as traffic generator. Destination of packets: Measurement probe acting as recipient. Underlying SUT: Network components between the source and destination. Measurement conducted at layer: Application. Complementary measurement are collected at lower layers.		
**Methodology**: For measuring UDP Throughput, a packet stream is emitted from a source and received by a data sink (destination). The amount of data (bits) successfully transmitted per unit of time (seconds), as measured by the traffic generator, shall be recorded. A UDP-based traffic stream is created between the source and destination while using the iPerf2 tool. The test case shall include the consecutive execution of several iterations, according to the following properties: Duration of a single iteration: at least three minutes. Records throughput over one-second intervals within an iteration. Number of iterations (*I*): at least 25.		
**iPerf2 configuration:**		
**Parameter**	**iPerf option**	**Suggested value**
Throughput measurement interval	–interval	1
Number of simultaneously transmitting probes/processes/threads	–parallel	−4 (in order to generate high data rate in the source)
Bandwidth limitation set to above the maximum bandwidth available	–b	Depends on the maximum theoretical throughput available in the network
Format to report iPerf results	–format	m [Mbps]
**Calculation process and output:** Once the KPI samples are collected, time-stamped (divided by iteration), and stored, evaluate relevant statistical indicators, e.g., average, median, standard deviation, percentiles, minimum, and maximum values, as follows: Evaluate the statistical indicators separately for each iteration. Evaluate the average of each indicator over the iterations, thus obtaining that same indicator for the test case. Evaluate and add a Confidence Interval (CI) * to the obtained indicator, which denotes the precision of the provided outcome. * A 95% CI is suggested and it should be evaluated while assuming a Student T-distribution for the data sample, with I−1 degrees of freedom. This accounts for non-Gaussian distributions of values collected at each iteration.		
**Complementary measurements (if available)**: Throughput at Packet Data Convergence Protocol (PDCP) and Medium Access Control (MAC) layers, Reference Signal Received Power (RSRP), Reference Signal Received Quality (RSRQ), Channel Quality Indicator (CQI), Adopted Modulation, Rank Indicator (RI), Number of MIMO layers, MAC, and Radio Link Control (RLC) Downlink Block Error Rate (BLER). For each measurement (or selected ones), provide the average per iteration and for the entire test case, following the procedure in “Calculation process and output”. **Note**: packet loss rate is not recorded because constant traffic in excess of the available capacity will be injected and the excess will be marked as lost packets, as could be expected.		
**Preconditions**: The scenario has been configured. In case of network slicing, the slice must be activated. The traffic generator should support the generation of the traffic pattern defined in “Methodology”. Connect a reachable UE (end point) in the standard 3GPP interface SGi or N6 (depending on whether the UE is connected to EPC or 5GC, respectively). Deploy the monitoring probes to collect throughput and complementary measurements. Ensure that, unless specifically requested in the scenario, no unintended traffic is present in the network.		
**Applicability**: The measurement probes should be capable of injecting traffic into the system as well as determining the throughput of the transmission.		
**Test case sequence**: Start monitoring probes. Start the traffic generator for transmitting from the client to the server probe, as described in “Methodology”. Record the throughput for each time interval within a trial. Stop the traffic generator. Stop monitoring probes. Calculate and record the KPIs as needed per iteration as defined in “Calculation process and output”. Repeat steps 1 to 6 for each one of the 25 iterations. Compute the KPIs as defined in “Calculation process and output”.		

**Table 3:** Scenario template applied to a 5G NR NSA deployment with Line of Sight (LoS) between gNB and UE. The scenario (SC) is denoted SC_LoS_PS, where PS stands for Proactive Scheduling, a vendor-specific configuration available in the gNB (cf. Section 5.3 for more details).

Scenario ID	SC_LoS_PS
Radio Access Technology	5G NR
Standalone/Non-Standalone	Non-Standalone
LTE to NR frame shift	3 ms
Cell Power	40 dBm
Band	n78
Maximum bandwidth per component carrier	40 MHz
Subcarrier spacing	30 kHz
Number of component carriers	1
Cyclic Prefix	Normal
Number of antennas on NodeB	2
MIMO schemes (codeword and number of layers)	1 CW, 2 layers
DL MIMO mode	2 × 2 Close Loop Spatial Multiplexing
Modulation schemes	256-QAM
Duplex Mode	TDD
Power per subcarrier	8.94 dBm/30 kHz
TDD uplink/downlink pattern	2/8
Random access mode	Contention-based
Scheduler configuration	Proactive scheduling
User location and speed	Close to the base station, direct line of sight, static
Background traffic	No
Computational resources available in the virtualized infrastructure	N/A

**Table 4:** Experiment 1: Statistical results of TC_THR_UDP test case in SC_LoS_PS and SC_NLoS_PS scenarios (Acronyms, as per Table 2; PDSCH MCS CW0 stands for Physical Downlink Shared Channel MCS for Codeword 0).

Parameter	Indicator	Scenario	
		LoS	NLoS
UDP Throughput [Mbps]	Average	179.45 ± 0.34	14.95 ± 0.45
	Median	179.14 ± 0.37	15.25 ± 0.09
	Min	162.86 ± 6.56	5.78 ± 1.65
	Max	195.80 ± 2.19	19.45 ± 3.34
	5% Percentile	171.22 ± 0.58	11.71 ± 1.00
	95% Percentile	188.64 ± 1.21	17.64 ± 2.53
	Standard deviation	5.47 ± 0.54	2.09 ± 0.78
SINR [dB]	Average	21.73 ± 0.07	7.27 ± 0.26
PDSCH MCS CW0	Average	27.00 ± 0.00	2.96 ± 0.02
RSRP [dBm]	Average	−52.42 ± 1.52	−115.41 ± 0.29
RSRQ [dB]	Average	−10.80 ± 0.00	−11.61 ± 0.04
PDSCH Rank	Average	2.00 ± 0.00	1.001 ± 0.001
MAC DL BLER [%]	Average	0.23 ± 0.05	8.77 ± 0.13

**Table 5:** Experiment 1: Statistical results for TC_RTT test case in SC_LoS_PS and SC_NLoS_PS scenarios (Acronyms as per Table 2; ReTx in MAC UL ReTx Rate stands for Retransmission).

Parameter	Indicator	Scenario	
		LoS	NLoS
RTT [ms]	Average	11.83 ± 0.05	26.15 ± 3.87
	Median	11.77 ± 0.07	19.33 ± 0.39
	Min	10.00 ± 0.00	13.19 ± 0.13
	Max	16.65 ± 0.60	555.16 ± 620.11
	5% Percentile	10.13 ± 0.02	14.39 ± 0.14
	95% Percentile	13.70 ± 0.09	42.74 ± 8.27
	Standard deviation	1.20 ± 0.04	48.33 ± 8.27
SINR [dB]	Average	21.58 ± 0.02	10.68 ± 0.14
MAC UL ReTx Rate [%]	Average	0.01 ± 0.001	14.83 ± 1.12
RSRP [dBm]	Average	−53.53 ± 0.05	−110.38 ± 0.18
RSRQ [dB]	Average	−10.80 ± 0.00	−11.15 ± 0.01
MAC DL BLER [%]	Average	0.01 ± 0.01	0.04 ± 0.02

**Listing 1:** Instantiation of the Generic Network Slice Template. Throughput and Maximum Transfer Unit (MTU) values are given in kbps and bytes, respectively.

1	{
2	" base_slice_descriptor ": {
3	" base_slice_des_id ": " AthonetEPC ",
4	" coverage ": [" Campus "],
5	" delay_tolerance ": true ,
6	" network_DL_throughput ": {
7	" guaranteed ": 100.000
8	},
9	" ue_DL_throughput ": {
10	" guaranteed ": 100.000
11	},
12	" network_UL_throughput ": {
13	" guaranteed ":10.000
14	},
15	" ue_UL_throughput ": {
16	" guaranteed ": 10.000
17	},
18	" mtu ": 1500
19	},
20	}

**Listing 2:** ED Template.

1	{
2	ExperimentType : Standard / Custom / MONROE
3	Automated : <bool >
4	TestCases : <List [str ]>
5	UEs : <List [ str ]> UEs IDs
6	
7	Slices : <List [ str]>
8	NSs : <List [ Tuple [str , str ]]> ( NSD Id , Location )
9	Scenarios : <List [str ]>
10	
11	ExclusiveExecution : <bool >
12	ReservationTime : <int > ( Minutes )
13	
14	Application : <str >
15	Parameters : <Dict [str , obj ]>
16	
17	Remote : <str > Remote platform Id
18	RemoteDescriptor : <Experiment Descriptor >
19	
20	Version : <str >
21	Extra : <Dict [str ,obj ]>
22	}

**Listing 3:** ED for Experiment 1 (evaluation of throughput and latency KPIs in LoS vs. NLoS scenarios).

1	{
2	ExperimentType : Standard
3	Automated : Yes
4	TestCases : TC_THR_UDP , TC_RTT
5	UEs : UE_1
6	Slice : Default
7	Scenario : SC_LoS_PS , SC_NLoS_PS
8	ExclusiveExecution : yes
9	Version : 2.0
10	}

**Listing 4:** ED for Experiment 2 (evaluation of latency KPI with vs. without PS activated).

1	{
2	ExperimentType : Standard
3	Automated : Yes
4	TestCases : TC_RTT
5	UEs : UE_1
6	Slice : Default
7	Scenario : SC_LoS , SC_LoS_PS
8	ExclusiveExecution : yes
9	Version : 2.0
10	}

**Listing 5:** ED for Experiment 3 (evaluation of throughput KPI in two 5G NR devices, UE_1 and UE_2).

1	{
2	ExperimentType : Standard
3	Automated : Yes
4	TestCases : TC_THR_UDP
5	UEs : UE_1 , UE_2
6	Slice : Default
7	Scenario : SC_LoS_PS
8	ExclusiveExecution : yes
9	Version : 2.0
10	}
